# A Case of Congenitally Corrected Transposition of Great Arteries: An Infrequent Happenstance

**DOI:** 10.1155/2017/7565870

**Published:** 2017-02-09

**Authors:** Prakash Ajmera, Vikas Medep

**Affiliations:** NH Malla Reddy Narayana Multispeciality Hospital, Hyderabad, Telangana 500055, India

## Abstract

Congenitally corrected transposition of the great arteries (CCTGA) is rare form of congenital heart diseases. It may be present with or without associated anomalies. Patients with CCTGA are usually diagnosed at early stages of life due to associated anomalies, but they may even remain asymptomatic till later decades of their life. We report a case of a 42-year-old man who presented at neurosurgery department with dizziness, seizures, and loss of consciousness, in whom isolated CCTGA was discovered incidentally. Further investigation depicted right ventricular hypertrophy, mild valvular regurgitation, mildly dilated pulmonary artery, low heart rate with AV dissociation, and third-degree heart block. These indicated for implantation of permanent pacemaker into the patient. The implantation of VVI mode pacemaker was uneventful and the patient is being followed up in the past eight months in favorable condition.

## 1. Introduction

Congenitally corrected transposition of the great arteries (CCTGA) is characterized by transposed great arteries and inverted ventricles, atrioventricular valves, and conduction system but normal atrial situs. It is a rare abnormality, occurring in approximately 0.5 to 1.4% of all congenital heart diseases [1, 2]. Merely 10% of the patients with CCTGA do not have any associated anomalies like ventricular septal defect, pulmonary artery stenosis, tricuspid valve abnormalities, and mitral valve abnormalities [3].

The patients without any associated anomalies, that is, isolated CCTGA, remain asymptomatic for many years and are usually diagnosed in later decades of life due to abnormal electrocardiograph (ECG), cardiomegaly on chest X-ray, or murmur [4]. Echocardiography, cardiac computed tomography, and cardiac magnetic resonance imaging (MRI), and electrocardiography are successfully utilized for meticulous diagnosis [1].

However, these patients are not devoid of complications. They often present with life threatening complications like systemic ventricular dysfunction, tricuspid regurgitation, heart block, and ventricular arrhythmia [5]. These lead to increased morbidity and mortality rates in such patients. By and large, the patients without associated anomalies have a normal life expectancy, but their lifespan markedly depends on the systemic ventricular function [6].

We present a case of incidental detection of isolated CCTGA in a patient in his fourth decade of life, who presented with dizziness, seizures, and loss of consciousness. After further investigation, he was managed successfully with VVI mode permanent pacemaker implantation and his condition is favorable on subsequent follow-ups.

## 2. Case Report

A 42-year-old male, occasional drinker, presented to neurosurgery department with complaint of dizziness, seizures, and loss of consciousness since 20 days. He was afebrile, nonhypertensive, and nondiabetic. His hematological, serological, and biochemical investigations were found normal, except for raised SGPT (ALT) enzyme (62 IU/L). Thereby, further investigations were performed.

**Figure 1:**
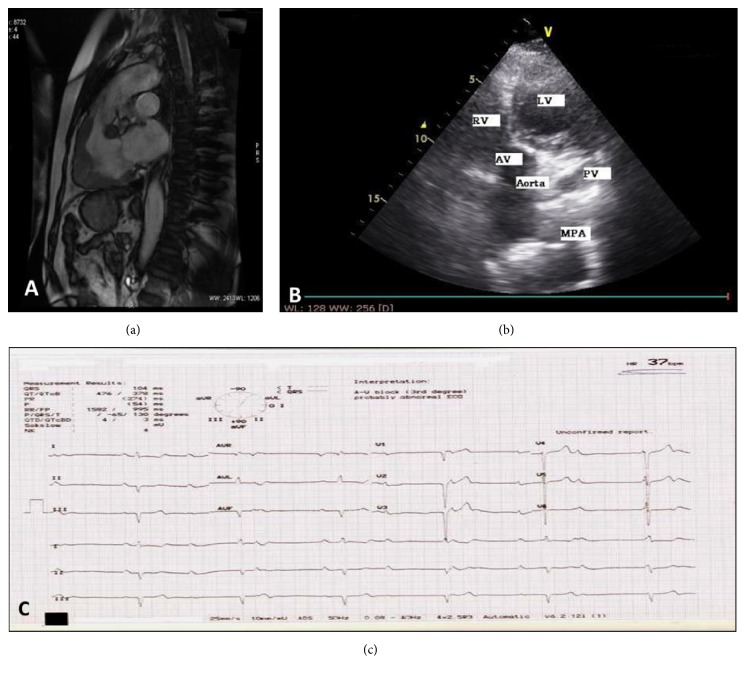
(a) Cardiothoracic MRI depicting right ventricular hypertrophy; (b) echocardiography image demonstrating transposed ventricles; (c) electrocardiogram interpreting AV block and low heart rate.

Magnetic Resonance Imaging was done, which showed morphological left ventricle (LV) on right side, small in caliber; morphological right ventricle (RV) on left side with myocardial hypertrophy; aorta rising from RV; and mildly dilated pulmonary artery rising from LV (Figure 1(a)). Findings of MRI were of concern for echocardiography. 2D echocardiography established the presence of congenitally corrected transposition of great arteries (CCTGA) (Figure 1(b)). Findings of echocardiography correlated with MRI. Moreover, grade 1 left ventricular diastolic dysfunction, mild pulmonary regurgitation, mild tricuspid regurgitation (TR), mild mitral regurgitation, and low heart rate with AV dissociation were noted during echocardiography. Ejection fraction was 64%. Electrocardiography depicted third-degree atrioventricular block (AV block) and heart rate of 37 beats per minute (Figure 1(c)).

**Figure 2:**
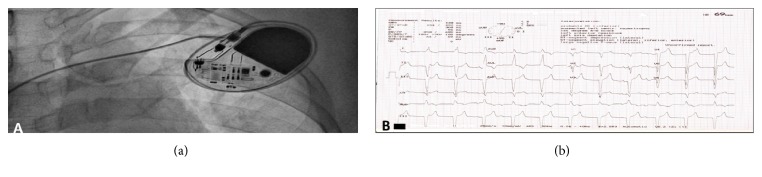
(a) Image showing implanted permanent pacemaker; (b) electrocardiogram after implantation of pacemaker.

CCTGA with third-degree AV block, right ventricular hypertrophy, and low heart rate indicated implantation of permanent pacemaker into the patient. Permanent pacemaker implantation of VVI mode was done through left subclavian approach under local anesthesia (Figure 2(a)). Extrathoracic subclavian vein puncture was done. A 58-1888 screw-in lead was placed in the left ventricular apex. A pulse generator was connected. Lead parameters were acceptable. The incision was closed in layers after checking the lead position. Procedure was uneventful and well tolerated by patient. The placement of pacemaker reverted normal heart rate and postimplantation ECG depicted AV block dissociation (Figure 2(b)). His condition is favorable at eight months' follow-up.

## 3. Discussion

Isolated CCTGA occur without any associated anomalies but can also be accompanied with complications. In our case, the patient was asymptomatic concerning the presence of CCTGA, which was unpremeditatedly diagnosed when the patient presented at neurosurgery department with unusual symptoms like dizziness, seizures, and loss of consciousness. However, further diagnosis majorly revealed mild valvular regurgitation, mild pulmonary artery dilatation, right ventricular hypertrophy (RVH), low heart rate, and third-degree AV block. By and large, the symptoms commonly associated with occurrence of TR in CCTGA are decreased stamina, dyspnea, chest pain, and palpitations [7]. Regardless of this, the patient did not present with any of these symptoms of TR or AV block; instead the dizziness and loss of consciousness have been allied with AV block.

In about 20 to 50% of CCTGA patients, TR occurs in varying degrees ensuing substantial morbidity and mortality. It has also been implicated as a cause of ventricular dysfunction. But it does not wholly explain the divergence of RV size and function [7]. Usually with increasing age, these patients present with atrial fibrillation and flutter [4], but in our case the patient experienced low heart rate. Every year around 2% of patients with CCTGA acquire complete AV block [6]. Displacement of AV node and abnormal course of conduction tissue upsurges the risk of development of AV block [8].

Recently, Zimmermann et al. had reported a case of CCTGA that was diagnosed on presentation of patient with acute myocardial infarction. On detailed diagnosis, he demonstrated isolated CCTGA with systolic dysfunction and ejection fraction of 30% [3]. He was managed with dual-chamber implantable cardioverter defibrillator. Similarly, Tandon et al. also reported an incidental diagnosis of dextroversion CCTGA in a patient who presented with cocaine-induced acute myocardial infarction [1]. However, in this case the patient did not present with specified cardiac complaints; instead he was primarily presented at the neurosurgery department, and then on referral for further investigation CCTGA was diagnosed.

Patients with relatively complicated CCTGA eventually require cardiac transplantation; while some patients can be managed with pacemaker implantation. More often than not, pacemaker implantations in such settings have been technically challenging pertaining to the complex heart anatomy [9]. Moreover, pacemaker implantation in such patients might lead to worsening of systemic ventricular function and atrioventricular valve regurgitation. The reason behind this is probably the modification in position of ventricular septum that induces a septal shift and failure of tricuspid valve coaptation [10]. Irrespective of the above complications, pacemaker was successfully implanted in our patient and is being followed up in the past eight months in favorable condition.

## 4. Conclusion

This was a rare case presentation as the patient remained asymptomatic for a long time (42 years); moreover, it was an accidental diagnosis of CCTGA accompanied with AV block. The placement of pacemaker reverted normal heart rate. We need to put screw-in lead as anatomical right ventricle is left ventricle as cavity will be smooth. Most of the cases inferior vena cava interruption occurs, so we need to prepare for hemiazygos route.
